# Clinical Significance of Cerebral Microbleeds Locations in CADASIL with R544C NOTCH3 Mutation

**DOI:** 10.1371/journal.pone.0118163

**Published:** 2015-02-18

**Authors:** Jung Seok Lee, Chul-hoo Kang, Sukh Que Park, H. Alex Choi, Ki-Bum Sim

**Affiliations:** 1 Department of Neurology, Jeju National University Hospital, Jeju National University College of Medicine, Jeju, South Korea; 2 Department of Neurosurgery, Soonchunhyang University Seoul Hospital, Soonchunhyang University College of Medicine, Seoul, South Korea; 3 Department of Neurosurgery and Neurology, University of Texas Medical School at Houston, Houston, Texas, United States of America; 4 Department of Neurosurgery, Jeju National University Hospital, Jeju National University College of Medicine, Jeju, South Korea; University of Glasgow, UNITED KINGDOM

## Abstract

**Background and Purpose:**

Although cerebral autosomal dominant arteriopathy with subcortical infarcts and leukoencephalopathy (CADASIL) is the most common single-gene disorder of cerebral small blood vessels caused by NOTCH3 mutations, little has been described about the variation in the clinical findings between its underlying types of mutations. In particular, the presence of cerebral microbleeds (CMBs) has been an increasingly recognized magnetic resonance imaging finding in CADASIL, but their clinical significance is not clear. The purpose of this study is to assess whether CMBs are associated with symptomatic stroke in the CADASIL patients with R544C mutation and to compare the cerebral distribution of CMBs between CADASIL patients with and without symptomatic stroke.

**Methods:**

This is a cohort study of patients who were diagnosed with genotype-confirmed R544C-mutation CADASIL. Primary neurologic symptoms were recorded. Symptomatic strokes were defined as transient ischemic attack, ischemic strokes and hemorrhagic strokes. CMBs were defined as focal areas of round signal loss on T2*-weighted gradient echo planar images with a diameter of less than 10 mm. The locations of CMBs were divided into lobar, basal ganglia, thalamus, brain stem and cerebellum. Multiple logistic regressions were performed to identify the epidemiologic or vascular risk factors associated with symptomatic stroke in patients with CADASIL.

**Results:**

Among total of 51 subjects in this cohort, CMBs were present in 20 of 32 patients (64.5%) in the symptomatic stroke-group and in 8 of 19 patients (42.1%) in the non-stroke group (p = 0.16). CMBs were observed more frequently in the basal ganglia (p<0.001) and the cerebellum (p<0.018) in the symptomatic stoke group compared to the non-stroke group. The mean number of CMBs was significantly higher in the symptomatic stroke group (15.4±18.0 lesions per patients with CMBs) versus those without symptomatic stroke (3.3±3.0 lesions per patients with CMBs) (p = 0.003). Hypertension was an independent risk factor for symptomatic stroke in CADASIL (p = 0.014). It was independently associated with CMBs locations as basal ganglia (p = 0.016), thalamus (p = 0.010), brainstem (p = 0.044), and cerebellum (p = 0.049). However, It was not independently associated with CMBs on lobar lesion (p = 0.152).

**Conclusions:**

In this study hypertension was an independent predictor of CMBs presence in specific brain locations, as well as symptomatic stroke in the CADASIL patients. The distribution and burden of CMBs might be a clinically useful marker for the risk of symptomatic stroke. However, further prospective studies on the relationship between CMBs distribution and symptomatic stroke are required in order to support these preliminary findings.

## Introduction

Cerebral autosomal dominant arteriopathy with subcortical infarcts and leukoencephalopathy (CADASIL) is a hereditary disease of the small blood vessels caused by mutations in the NOTCH3 gene.[1] Although the mutations are highly stereotyped, clinical phenotypes are variable, stressing the importance of studying populations of patients with specific mutations.[2] The main clinical manifestations are recurrent stroke, cognitive decline, chronic headache, mood disturbances, and seizure.[3,4] Magnetic resonance imaging (MRI) is crucial in the diagnosis of CADASIL. Typical MRI findings include multiple subcortical lacunes, extensive white matter change, and multiple cerebral microbleeds (CMBs).[5] CMBs are well defined as pathological lesions that can be detected with the use of T2*-weighted gradient echo,[6] and have been highlighted as markers for and contributors to CADASIL.[7] Although some studies have shown that the presence of CMBs predict recurrence of ischemic stroke as well as hemorrhagic stroke,[7] the clinical significance of the CMBs observed in CADASIL has not been clearly elucidated.

We sought to examine the associations of CMBs and the presence of symptomatic stroke and to compare the cerebral distribution of CMBs between R544C mutation CADASIL patients with and without symptomatic stroke.

## Methods

Subjects were drawn from an ongoing prospective cohort study of patients with CADASIL on Jeju Island in Korea. Subjects were recruited from consecutive CADASIL patients. They were all at least 18 years of age, evaluated at Jeju National University Hospital between April 2008 and December 2009, with diagnosis confirmed by the identification of an R544C mutation in the NOTCH3 gene. Patients who were pregnant or had other contraindications for MRI were excluded. Asymptomatic subjects (n = 7) were excluded. This study was approved by the Institutional Review Board (Jeju National University Hospital Institutional Review Board), and written informed consent was obtained from all patients.

The vascular risk factors were recorded, including hypertension, diabetes mellitus, and hypercholesterolemia. Hypertension was defined as blood pressure > 140/90 mmHg on different occasions or use of an antihypertensive agent. Diabetes mellitus was defined as fasting glucose level ≥ 126 mg/dl or PP2 test level ≥ 200 mg/dl or use of antidiabetes medication. Hypercholesterolemia was defined as total serum cholesterol level > 240mg/dl.

The final study population consisted of 51 patients. Patients were divided into two groups, depending on the presence (symptomatic stroke group; n = 32) or absence (non-stroke group; n = 19) of symptomatic stroke presentation. Symptomatic stroke was defined as subjects with a history of an episode of neurological dysfunction caused by focal ischemic injury (TIA, cerebral infarction) or focal collection of blood within brain parenchyma that is not caused by trauma (ICH).[9] All ischemic and hemorrhagic strokes were confirmed by brain imaging confirmation. Information about the history of TIA and stroke was collected through semi-structured interviews of the patients or family. Then we reviewed the medical records.

**Fig 1:**
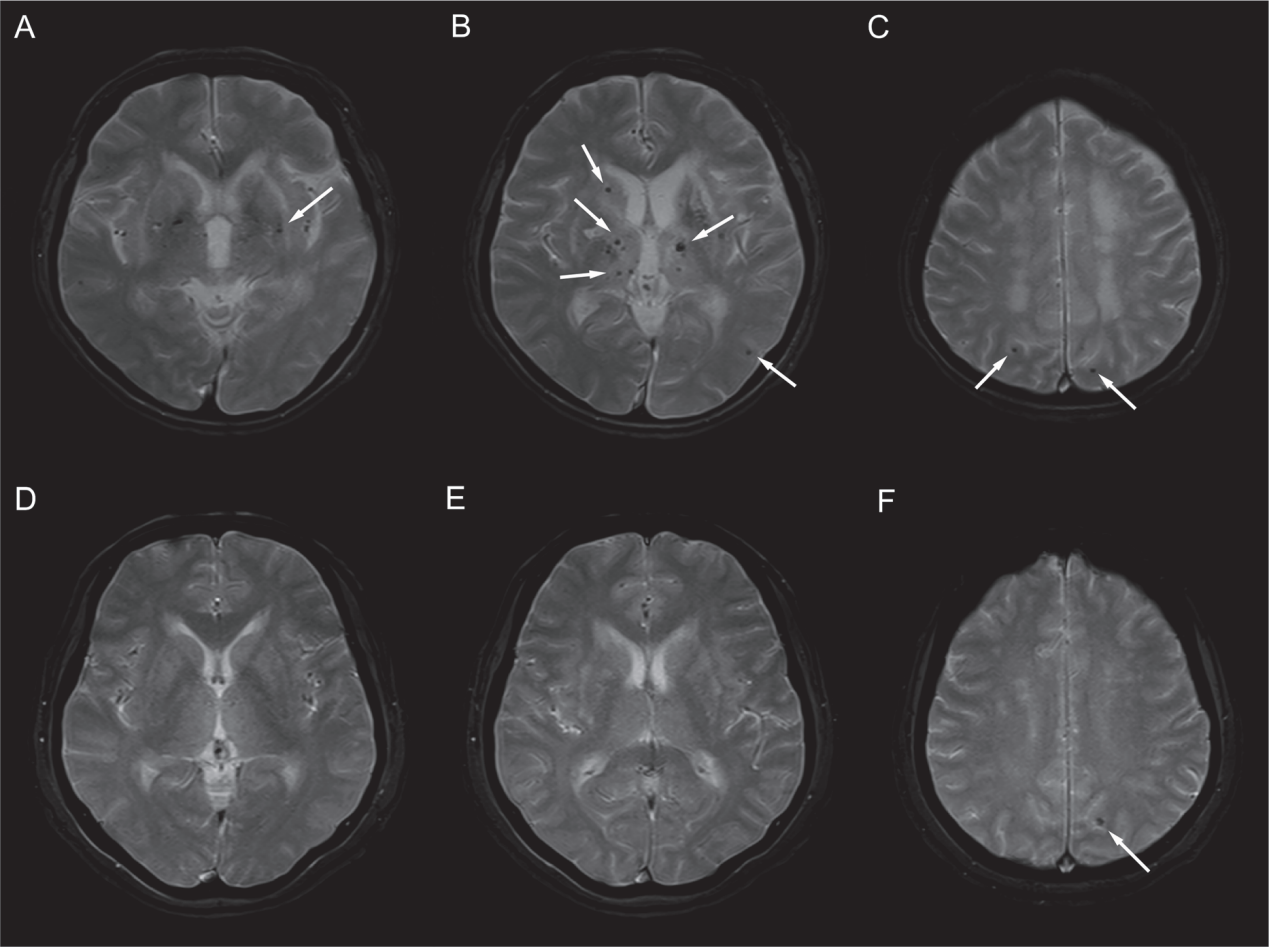
T2*-weighted gradient image of a patient (aged 66 years) with clinically overt stroke (A-C). Multiple cerebral microbleeds (CMBs) were observed in the basal ganglia and thalamus (A, B). In addition, CMBs were observed in subcortical areas in both parietal lobes (C). T2*-weighted gradient image of a patient (aged 62 years) without clinically overt stroke (D-E). A single cerebral microbleed was observed in subcortical areas in the left parietal lobe (E).

MRI studies were done using a 1.5-tesla-system (Sonata; Siemens, Erlangen, Germany). The brain imaging protocol included the following (all protocols used a slice thickness of 5 mm and an inter-slice gap of 1.5 mm): T1-weighted images (time to echo, TE = 9.3 ms and time to repeat, TR = 550ms), T2 *-weighted gradient echo planar images (TE = 20 ms and TR = 600 ms), and FLAIR (fluid-attenuated inversion recovery) images (TE = 135 ms and TR = 8,100 ms). CMBs were defined as focal areas of round signal loss on T2*-weighted gradient echo planar images with a diameter of less than 10 mm (Fig. 1). The total number of CMBs was counted manually by two observers according to current consensus criteria.[10] The location of CMBs was categorized into separate cerebral region on the basis of previous studies.[11]

Lacunes were defined as parenchymal defects not extending to the cortical gray matter with a signal intensity of cerebrospinal fluid in all sequences and more than 2 mm in diameter. Lesions located in the lower third of the corpus striatum of the basal ganglia were excluded. White-matter hyperintensities (WMHs) were defined as white-matter areas with increased signal intensities on FLAIR images. All FLAIR axial sections from the base of the cerebellum to the vertex were analyzed. A masking and thresholding technique was used (Analyze 8.1, Biomedical Imaging, Mayo Clinic, Rochester, MN, USA). The total volume of WMHs was calculated automatically by multiplying the lesion area by the section thickness. WMH volume was normalized for total brain volume by dividing the individual WMH volume by the intracranial cavity volume [normalized WMH volume (nWMH)]. The imaging analysis (presence of lacunes, presence of CMBs) were performed by two experienced neurologist (J.S.L., C.K.) operating by consensus and without any knowledge of clinical information. First, the presence, total number and locations of CMBs were analyzed. Then, the locations of CMBs were categorized into separate cerebral regions on the basis of common locations of hemorrhage in CADASIL and on the basis of previous studies. These regions were classified as follows: 1) lobar 2) basal ganglia 3) thalamus 4) brain stem and 5) cerebellum. The significance of the presence of CMBs according to the location was analyzed.

Data were analyzed using SPSS statistical software (version 20.0). We used the Mann-Whitney test, as appropriate; to compare the epidemiologic and radiologic data between the two groups (patients with or without symptomatic stroke). The χ^2^ test or Fischer’ exact test was used for analysis for categorical variables. Variable with a p-value of less than 0.20 on univariate analysis were included in the multivariated model to identify the factor associated with symptomatic stroke in patients with CADASIL. Odds Ratios (OR) for factors associated with symptomatic stroke were calculated using a multivariable logistic regression analysis that included hypertension, presence of lacunes, presence of CMBs, and nWMHs. The associations were expressed as ORs with 95% continuous interval (CI). We used the Pearson χ2 test or Fisher exact test to compare the frequency distribution of CMBs between two groups. The Mann-Whitney U test and χ^2^ analyses were also used for group comparisons (patients with and without CMBs). The independences of relationships between hypertension and different CMBs location (including lobar area, basal ganglia, thalamus, brainstem, and cerebellum) were tested by multivariate logistic regression analysis. Variables with p-values less than 0.20 on univariate analysis were included in the multivariable model. A p value of 0.05 or less was defined as statistically significant.

## Results

Details of demographics and MRI findings of patients with and without symptomatic stroke are presented in Table 1. Of the 51 patients, 27 were men (52.9%). The mean age of the patients was 62.5±11.6 years (range 31–81 years). Symptomatic stroke presentation was the most frequent manifestation (n = 32), followed by chronic headache (n = 18), dementia (n = 6), Parkinsonism (n = 4), and seizure (n = 2). Symptomatic stroke included patients with ischemic stroke (n = 25), TIA (n = 7), or ICH (n = 4). Hypertension was present in 26 patients with R544C mutation CADASIL (51%).

**Table 1:** Demographic and clinical characteristics of CADASIL in patients with the R544C mutation.

	No stroke (n = 19)	Stroke (n = 32)	p value
Demographics			
Age, y (SD)	59.8±11.7	64.1±11.4	0.254
male, female	8, 11	19, 13	0.232
Education, y (SD)	9.1±4.9	7.4±5.8	0.355
Medical history			
Hypertension	4 (21.0)	22 (68.7)	0.001
Diabetes Mellitus	2 (10.5)	4 (11.4)	1.000
Hypercholesterolemia	1 (5.3)	7 (21.9)	0.231
Ever-smoking	5 (26.3)	10 (31.3)	0.688
Ischemic heart disease	1 (5.3)	3 (9.4)	1.000
MRI characteristics			
Presence of lacunes	8 (42.1)	25 (78.1)	0.009
Number of lacunes	2.5±5.6	4.5±6.4	0.016
Presence of CMBs	8 (42.1)	20 (62.5)	0.157
Number of CMBs	1.4±2.5	9.9±16.1	0.010
nWMHs	2.3±2.0	3.1±1.5	0.037

Data are mean ± SD or n (%) values.

*CADASIL* cerebral autosomal dominant arteriopathy with subcortical infarcts and leukoencephalopathy

*nWMHs* normalized White Matter Hyperintensities.

CMBs were observed in 54.9% of the total patients. The average number of CMBs was 11.9 lesions per patient. CMBs were found in 20 of 32 patients (62.5%) in the symptomatic stroke group and in 8 of 19 patients (42.1%) in the non-stroke group (p = 0.15). The most frequent locations of CMBs were the thalamus (47.1%), lobar (35.3%), and basal ganglia (29.4%). The mean number of CMBs was significantly higher in the symptomatic stroke group (15.4±18.0 lesions per patients with CMBs) versus those without symptomatic stroke (3.3±3.0 lesions per patients with CMBs) (p = 0.003).

The symptomatic stroke group showed a higher prevalence of CMBs in the basal ganglia (p<0.001) and the cerebellum (p = 0.018) than did the non-stroke group (Table 2). Five ICHs were found in four patients (7.8%) in the symptomatic stroke group. Two ICHs were located in the basal ganglia, two were in the temporal lobe, and one was in the cerebellum.

**Table 2:** Frequency of CMBs according to presence of symptomatic stroke.

Location	No stroke (n = 19)	Stroke (n = 32)	p-value
Any location			
Lobar	5 (26.3)	13 (40.6)	0.264
Basal ganglia	0 (0)	15 (46.9)	<0.001
Thalamus	6 (31.6)	18 (56.3)	0.069
Brainstem	3 (15.8)	9 (28.1)	0.332
Cerebellum	0	9 (28.1)	0.009

n (%) value

*CMBs* cerebral microbleeds

Hypertension (odds ratio and 95% Confidence interval: 6.78, 1.48–31.13) was an independent risk factor for symptomatic stroke in R544C mutation CADASIL (Table 3). There were statistically significant differences in prevalence of hypertension and number of lacunes between patients with and without CMBs (Table 4).

**Table 3:** Logistic Regression Results for predicting symptomatic stroke.

Variables	OR	95% CI	p-value
Hypertension	6.78	1.48–31.13	0.014
presence of lacunes	0.31	0.06–1.55	0.154
presence of CMBs	1.20	0.27–5.25	0.812
nWMHs	1.01	0.63–1.59	0.985

*CMB* cerebral microbleeds; *OR* odds ratio; *CI* confidence interval.

*nWMHs* normalized white matter hyperintensities

**Table 4:** Baseline characteristics of patients classified according to presence or absence of CMBs.

	No CMBs (n = 23)	CMBs (n = 28)	p value
Demographics			
Age, y (SD)	60.1±12.0	64.4±11.0	0.244
male, female	12, 11	15, 13	0.921
Medical history			
Hypertension	6 (26.1)	20 (71.4)	0.001
Diabetes Mellitus	1 (4.3)	5 (17.9)	0.204
Hypercholesterolemia	2 (8.7)	6 (21.4)	0.269
Ever-smoking	7 (33.3)	8 (29.6)	0.784
Ischemic heart disease	1 (4.3)	3 (10.7)	0.617
MRI characteristics			
Presence of lacunes	12 (52.2)	21 (75.0)	0.090
Number of lacunes	1.3±1.8	5.8±7.6	0.020
nWMHs	2.5±2.0	3.0±1.6	0.191

*CADASIL* cerebral autosomal dominant arteriopathy with subcortical infarcts and leukoencephalopathy

Data are mean ± SD or n (%) values.

In the multivariate logistic regression model (Table 5), hypertension was independently associated with CMBs in the basal ganglia (p = 0.016), CMBs in the thalamus (p = 0.010), CMBs in the brainstem (p = 0.044), and CMBs in the cerebellum (p = 0.049). However, hypertension was not independently associated with CMBs in the lobar lesion (p = 0.152). Age was independently associated with CMBs in the cerebellum (p = 0.025). There was a trend toward positive correlation between age and presence of CMBs in the brainstem (p = 0.059). When our patients had a history of hypertension or CMBs in the basal ganglia, the positive predictive value for symptomatic stroke increased to 78%.

**Table 5:** Logistic Regression Results for predicting CMBs according to cerebral location.

Variables	Location of CMBs	OR	95% CI	p-value
Hypertension	Lobar	2.63	0.70–9.91	0.152
Age	Lobar	1.03	0.97–1.09	0.315
Hypertension	Basal ganglia	6.55	1.41–30.40	0.016
Hypertension	Thalamus	5.95	1.52–23.3	0.010
Hypertension	Brainstem	5.49	1.05–28.81	0.044
Age	Brainstem	1.08	1.00–1.16	0.059
Hypertension	Cerebellum	11.85	1.10–138.9	0.049
Age	Cerebellum	1.14	1.02–1.28	0.025

*CMBs* cerebral microbleeds; *OR* odds ratio; *CI* confidence interval.

A total of 24 patients (47.0%) had CMBs in either the lobar area or deep structures of brain including basal ganglia and thalamus. In 18 R544C mutation CADASIL patients with symptomatic stroke, 15 patients had a mixed deep and lobar distribution of CMBs (83.3%) and 5 had isolated deep distribution of CMBs (27.8%). Only one patient had isolated lobar CMBs (5.6%). However, in 6 patients without symptomatic stroke, 3 patients had a mixed deep and lobar distribution of CMBs (50%) and 2 had isolated lobar distribution of CMBs (33.3%). Only one patient had isolated deep CMBs (16.7%).

## Discussion

We found that CMBs are common in CADASIL patients with R544C NOTCH3 mutation and the mean number of CMBs was significant higher in patients with symptomatic stroke. Patients with symptomatic strokes had significantly more CMBs in the basal ganglia and cerebellum compared to patients who did not have strokes. Although CMBs in the thalamus were relatively more frequent in patients with symptomatic stroke, it did not reach statistical significance. As CMBs are more easily detected by MR technology, population-based studies have shown CMBs to be common in community-dwelling elderly people, with prevalence between 10% and 25%. CMBs indicate specific underlying vascular pathological states, in particular hypertensive vasculopathy (for CMBs in deep hemispheric or infra-tentorial locations) or cerebral amyloid angiopathy (for CMBs restricted to lobar location). The presence and number of CMBs might also indicate the severity of these hemorrhage-prone pathological states and might also predict the risk of future symptomatic ICH.[10]

Not surprisingly, CMBs have also been observed in 31∼69% of CADASIL patients.[8,12] They are localized in several locations, preferentially in cortical-subcortical regions, white matter, thalamus and brainstem, suggesting that CMBs and ischemic lesions are manifestations of the same underlying angiopathy. Although genotype-phenotype correlations remain unclear in CADASIL,[5] studies suggest that there is a variation in clinical symptoms depending upon the specific mutation.[13, 14] For this reason, we studied CASASIL patients with only the R544C NOTCH3 mutation.

We found that 4 of the 32 patients with symptomatic stroke had ICH (12.5%). Previously Choi, et al, reported that the presence of CMBs was associated with ICH in the predominant R544C NOTCH3 mutation. The rate of ICH in this study was 12.3% (n = 9), with 8 symptomatic patients. All of the patients had CMBs.[15] In another study of 20 symptomatic patients with CADASIL, 7 ICHs in 5 patients was observed.[16] However, the studies did not address or describe the frequency or location of CMBs in detail.[15,16] In our study, the basal ganglia was the third most frequent location for CMBs (29.4%). Two of 5 ICHs with 4 patients were located in the basal ganglia (40%). Our results are consistent with reports showing that the predictive value for ICH was highest among the patients with advanced leukoaraiosis when CMBs were located in the basal ganglia.[17,18]

The cerebellum is known to be relatively preserved in patients with CADASIL. However, recent studies have shown that CMBs is detected in the cerebellum in 25% of cases [19] and ICH occurs in the cerebellum [16,20]. Choi, et al, reported that one of 7 ICHs with 5 patients was located in cerebellum (14.3%) [16]. In an Italian series, three patients with CADASIL developed ICH in the thalamo-capsular area and one of them had a recurrent cerebellar hemorrhage (25%) [20]. Our results suggest that CADASIL who have CMBs in the basal ganglia or the cerebellum may be at a higher risk for ischemic and hemorrhagic strokes. We also found that hypertension was an independent risk factor for symptomatic stroke in our CADASIL patients, and the thalamus and basal ganglia were the most common sites for CMBs in patients with symptomatic stroke. Only hypertension was found to be independent predictors of CMBs in the thalamus (p = 0.010) and CMBs in the basal ganglia (p = 0.016). However, hypertension was not associated with CMBs in the lobar lesion. Age (p = 0.025) and hypertension (p = 0.049) were independently associated with CMBs in the cerebellum. These findings support the hypothesis that hypertension is closely linked to CMBs in deep locations.[10]

Our study has several limitations. First, it was a cross-sectional study. Thus, further prospective studies are needed to elucidate the association between the presence of CMBs in specific locations and symptomatic stroke. Second, the number of patients was relatively small, which may have limited the statistical power to prove some correlations between hypertension and different CMBs location. Third, there may be some co-linearity among the independent variables to predict symptomatic stroke such as hypertension, presence of lacunes, presence of CMBs, and WMHs. Finally, the most severely disabled patients with the R544C mutation might have been excluded.

## Conclusions

In our study hypertension was found to be an independent predictor of CMBs in specific brain areas, as well as symptomatic stroke in CADASIL with R544C NOTCH3 mutation. A certain distribution and burden of CMBs might be a clinically useful marker for the risk of symptomatic stroke in these patients, but prospective cohorts are needed.
