# Correction: Clinical Significance of Cerebral Microbleeds Locations in CADASIL with R544C NOTCH3 Mutation

**DOI:** 10.1371/journal.pone.0125297

**Published:** 2015-04-09

**Authors:** Jung Seok Lee, Chul-hoo Kang, Sukh Que Park, H. Alex Choi, Ki-Bum Sim

There is an error in the Funding section of the published article. The Funding section should read: This study was supported by a grant of the Jeju National University Research Fund (2011). The funders had no role in study design, data collection and analysis, decision to publish, or preparation of the manuscript.
